# A Prospective Feasibility Study to Differentiate Sacral Neuromodulation Lead Electrode Configurations Using Motor and Sensory Thresholds and Locations of Sensation

**DOI:** 10.21203/rs.3.rs-4980674/v1

**Published:** 2024-10-18

**Authors:** Tianyu He, Christopher Hornung, Michael Evans, Stephanie Zoghbi, Leya Chahine, Fatima A. Nazar, Dwight Nelson, Nissrine Nakib

**Affiliations:** Department of Urology, University of Minnesota; Department of Urology, University of Minnesota; Clinical and Translational Science Institute, University of Minnesota; American University of Beirut; American University of Beirut; American University of Beirut; Department of Urology, University of Minnesota; Department of Urology, University of Minnesota

**Keywords:** Neurostimulation, Overactive Bladder, Sacral Neuromodulation, Motor Threshold, Sensory Threshold

## Abstract

**Background:**

Accurate positioning and effective programming of sacral neuromodulation (SNM) relies upon the use of several acute stimulation measurements. While the clinical utility of these acute measurements including pelvic floor motor thresholds (PFMT), toe/leg motor thresholds (TMT), and sensory thresholds (ST), are widely accepted, their usefulness in quantitative research remains unclear. The purpose of this prospective study was to test these measurements and gauge their utility in future research.

**Methods:**

Eight participants received Axonics SNM, 6 Medtronic Interstim II, and 2 Medtronic Micro SNM. PFMT was measured after implantation. ST and the location of sensation (LOS) were measured immediately postoperatively (PO), at pre-release from the surgery center (PR), and during a follow-up clinic visit (FU). Thresholds were compared across contact and time using linear mixed-effects models.

**Results:**

Significant differences in PFMT were found across electrode configurations, with stimulation through proximal contacts exhibiting lower PFMT than distal configurations. ST displayed no significant differences across electrodes and showed minimal changes over time. LOS exhibited substantial variability across patients and periods.

**Conclusions:**

Results suggest that PFMT were able to differentiate differences across electrode configurations that may be useful for future quantitative research. The lack of differences in ST and LOS across electrode configurations was interesting given the focus on these measurements clinically. Future testing is to confirm these limitations.

## Background

Sacral neuromodulation (SNM) is an effective therapeutic option for conditions such as overactive bladder, idiopathic urinary retention, and fecal incontinence.^1^ Optimal SNM therapy requires the precise implantation of an electrical lead with electrodes (or contacts) to modulate the sacral nerves.^1–3^ Throughout implantation and follow-up, standard-of-care measurements are made using acute responses to stimulation. Despite the acknowledged clinical value of these measurements, we conducted a feasibility study to investigate their potential as quantitative metrics capable of discerning subtle differences across lead contacts. The goal was to determine whether these measurements could quantify scientific-engineering therapy needs, such as optimal electrode locations or reduced lead migration, which are crucial for advancing SNM technologies.

Despite being a clinically significant therapy, there is a perennial call for research to improve and optimize SNM.^1, 4–6^ Evidence from preclinical and clinical studies point to small changes in lead position or contact location along the lead that may be visible in PFMT and ST. These findings support the hypothesis that small differences in the position of SNM lead contacts may be measurable through acute threshold testing.

During lead implantation and programming, acute responses to stimulation are used to target the nerve. Low PFMT responses (< 2 mA or < 2 V), in conjunction with fluoroscopy imaging, are used as indicators of proper implantation location relative to the sacral nerve.^1, 2, 7^ Immediately post-surgery, the lowest ST and the location of sensation (LOS)^8, 9^ are used to select therapy electrode configurations. These ST and LOS measurements are also used in subsequent clinical assessments to confirm therapy loss and potential lead migration, prompting reprogramming to alternative electrode configurations or possible lead revision.^3, 10^

There is abundant preclinical evidence suggesting that differences in implanted lead contact location affect sacral nerve activation. PFMT and similar motor responses identified contact locations optimally positioned near the sacral nerve. In sheep studies, lead contacts 0 and 1, positioned more proximally within the ventral sacral plane, were more effective for inducing motor responses than their more distal counterparts.^11^ Proximal contacts were also more effective for increasing bladder filling. These patterns hold true in more comprehensive tests in pigs and sheep.^12,13^

Similar findings are seen in human data. In cadaveric lead implantations, SNM contacts exhibit a range of contact-to-nerve distances where they are smaller for contacts proximal along the SNM lead, compared to more distal contacts.^8^ In human clinical testing, bipolar electrode configurations with cathodic stimulation delivered proximally on the leads (3−, 1 + and 3−, 0+) are associated with significantly larger pelvic floor motor responses and visibly lower thresholds than those with more distal cathode placements (0−, 3+; 1−, 3+; 2−, 3+).^14^ In addition to contact locations impacting effective responses to stimulation, lead movement or migration after the implant has also been described,^15,16^ and is associated with ST changes visible during (re)programming. Combined with therapy loss, ST changes are indicators of lead movement^17^.

This prospective clinical study is a feasibility test of these acute threshold measurements and their potential utility for future research. The primary hypothesis posited that PFMT, ST, and LOS would exhibit differences across contacts and change over time, addressing the question: Can these measurements effectively differentiate lead contact differences that may be present following implantation or post-surgical lead migration?

## Methods

**Figure 1:**
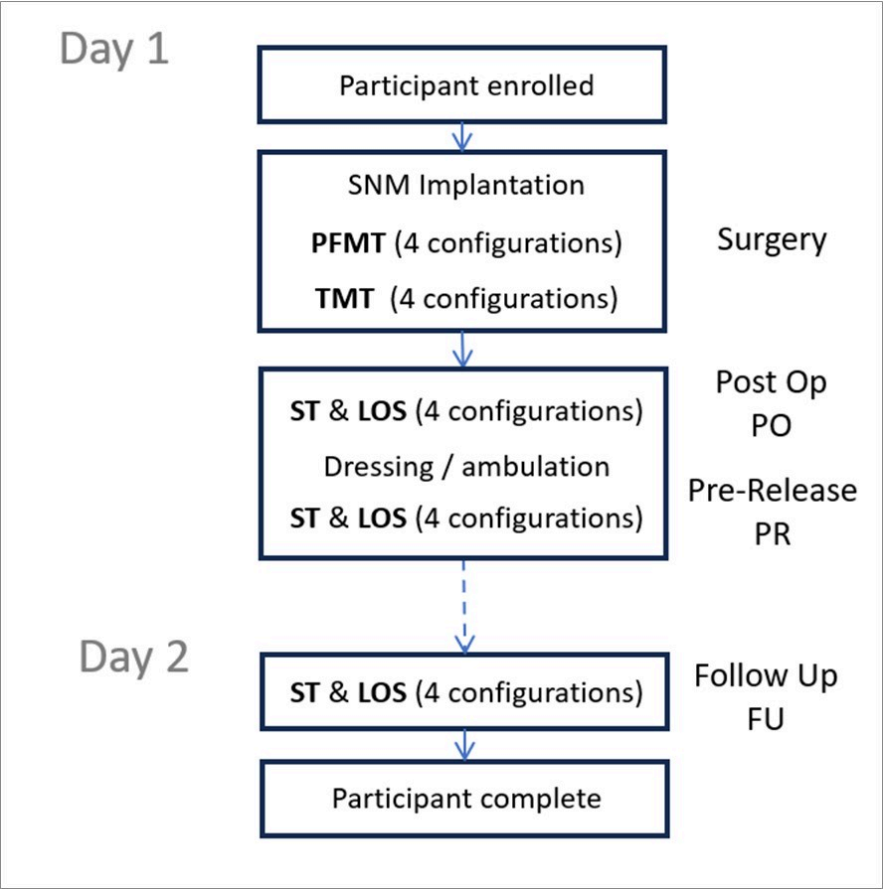
Design for this prospective clinical feasibility study. Participants were enrolled on the day of surgery and completed SNM implantation along with PFMT, ST and LOS during surgery, in post op and again pre-release following dressing. Follow up tests of ST and LOS were conducted on a return clinic visit.

This study was approved by the University of Minnesota Institutional Review Board (IRB) under STUDY9976 in Dec 2020, and it conformed to all necessary ethical standards, including the Declaration of Helsinki. All participants provided informed consent prior to participation. As this was not a clinical trial, a formal clinical trial number is not applicable. Motor and sensory thresholds for all therapy contacts were measured on the day of surgery and during a follow-up clinical visit to facilitate comparisons across time and contacts (Fig. 1). To align with current clinical and surgical practices, we used bipolar stimulation configurations of 0−, 3+; 1−, 3+; 2−, 0+; and 3−,0+, chosen to focus cathodic neural activation at contacts 0, 1, 2, and 3, respectively, along the lead from proximal to distal, relative to the stimulator. Stimulations used constant pulse width (210 μs) and frequency (14 Hz).

During surgery, we tested patients sedated under light anesthesia. After satisfactory needle responses, permanent SNM leads were implanted with positions confirmed through fluoroscopy. PFMT and TMT were measured using the clinical programmer temporarily connected to the lead. The pelvic floor or bellows response was observed for PFMT, while TMT was determined by visible toe, foot, or ankle movements. Stimulation was delivered at increasing amplitudes to reach the motor threshold, and the procedure was repeated at the next configuration.

ST was measured with awake patients in a reclined position. Stimulations were again delivered with increasing amplitudes as patients were queried about sensations. Amplitudes that induced perception were recorded as ST. During ST tests, LOS was also recorded using a pelvic area map. Participants identified specific coordinates corresponding to areas including the anus (Anal), perianal region (Perianal), genital area (Genital), and leg or groin (Other) areas ^9^. This procedure was repeated at three time points: post-operatively (PO), pre-release from the OR (PR), and during the Follow Up clinical visit (FU).

Data was recorded and stored using REDCap on an Android tablet, and later uploaded to a secure, confidential Box database. For this study, both Axonics and Medtronic stimulators were used. Since Axonics stimulators deliver stimulation therapy in mA and Medtronic in voltage or current, we did not directly compare the stimulation levels across company platforms. For stimulation threshold level comparisons, PFMT and ST, requiring consistent units we used only current measurements from Axonics data. When comparing LOS or similar thresholds not requiring identical measurement units, we combined current and voltage-based data.

ST and PFMT were compared by contact and time period using linear mixed-effects models, with fixed effect terms for contact, time period, and contact-by-time interaction, as well as a random intercept for participants to account for within-participant correlation. Differences in variability across the lead contacts were examined using Levene’s test for equality of variances. Changes in LOS were summarized using frequencies and percentages of agreement and near-agreement across visits as well as Student’s t-tests for direct comparisons. Analyses were conducted using R version 4.2.2 (R Foundation for Statistical Computing, Austria).

## Results

Data were collected from 16 participants, 11 females and 5 males, at the University of Minnesota Clinics and Surgery Center (Minneapolis, MN) from June 2021 - July 2022. Eight participants were implanted with Axonics SNM, 6 with Interstim II, and 2 with Interstim Micro. To allow the grouping of identical electrical units for quantified thresholds (mA), threshold comparisons used only the 8 participant datasets with Axonics SNM for PFMT and ST. LOS comparisons included all 16 datasets. All procedures were completed without adverse events or significant discomfort.

**Figure 2:**
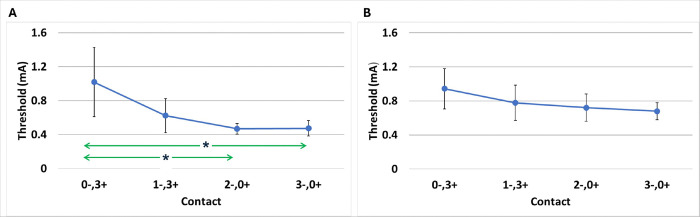
PFMT (A) and TMT (B) measured during surgical implant are shown for different contact stimulations. N=8 for PFMT; N=7 for TMT. PFMT was significantly smaller at proximal contact stimulation, 2−,0+ and 3−,0+, vs distal stimulation, 0−,3+ (P<0.05). For TMT (B) there were no significant differences across electrode configurations.

Post-surgical PFMTs were collected from all 16 enrolled participants, revealing a range between 0.4 and 3.6 mA across the electrode configurations (Fig. 2A). Significant differences were observed in PFMT across electrodes with distal (deep) contact cathodes (0−, 3+) having a significantly larger PFMT than proximal contact stimulations (2−, 0 + or 3−, 0+; P < 0.05). While distal PFMT configurations were visibly larger than proximal, variance across contacts was not significantly different (P > 0.05).

TMT ranged from 0.2 to 2mA across stimulation configurations (Fig. 2B). In contrast to PFMT, no significant differences were observed in TMT across contacts (P > 0.05). Furthermore, no significant differences were noted across different electrodes for these measurements (all pairwise comparisons, P > 0.05). While mean TMTs appeared larger than PFMTs across all electrode configurations (0.73+/−0.17, SEM, vs 0.64+/−0.17 mA, respectively), no differences were significant (P > 0.05 for both contact-specific differences and overall).

**Figure 3:**
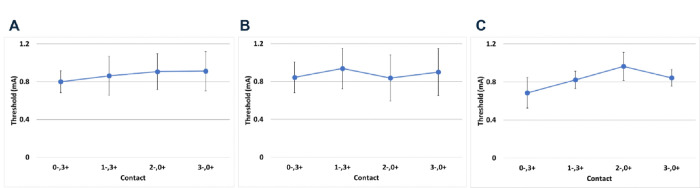
Mean ST (+/− SEM) measured across electrode configurations are shown for PO (A), PR (B) and FU (C). N=8 for PO and PR, N=7 for FU. There were no significant differences across electrode configurations within any of the time points. There was no significant difference between ST at PO vs PR, but there was a significant overall decrease in ST at PR vs FU (B vs C).

Post-surgical ST were collected at both the PO and PR time points from 15 of 16 enrolled participants, with ST collected in mA for the 7 participants with Axonics stimulators. For the FU time point, only 9 participants were tested and 8 of these were implanted with Axonics stimulators. The mean duration from PO to FU was 22d (SD = 7d; range = 8–29d). ST ranged between 0.05 and 2.6 mA across electrode configurations and time points tested (Fig. 3A–C). No significant differences were observed in ST across electrodes for any time point tested (PO, PR, or FU). There was also no significant difference in variability between distal and proximal electrodes for ST. While ST remained unchanged between PO and PR (P > 0.05; Fig. 3A–B), or FU and ST (P > 0.05; Fig. 3B–C), there was a statistically significant decrease from PR to FU (P < 0.05). The overall mean PFMT and ST (0.64+/−0.17 and 0.87+/−0.18 mA, respectively) were not different, nor was there a significant difference between these values across electrode configurations (P > 0.05 for all comparisons).

**Figure 4:**
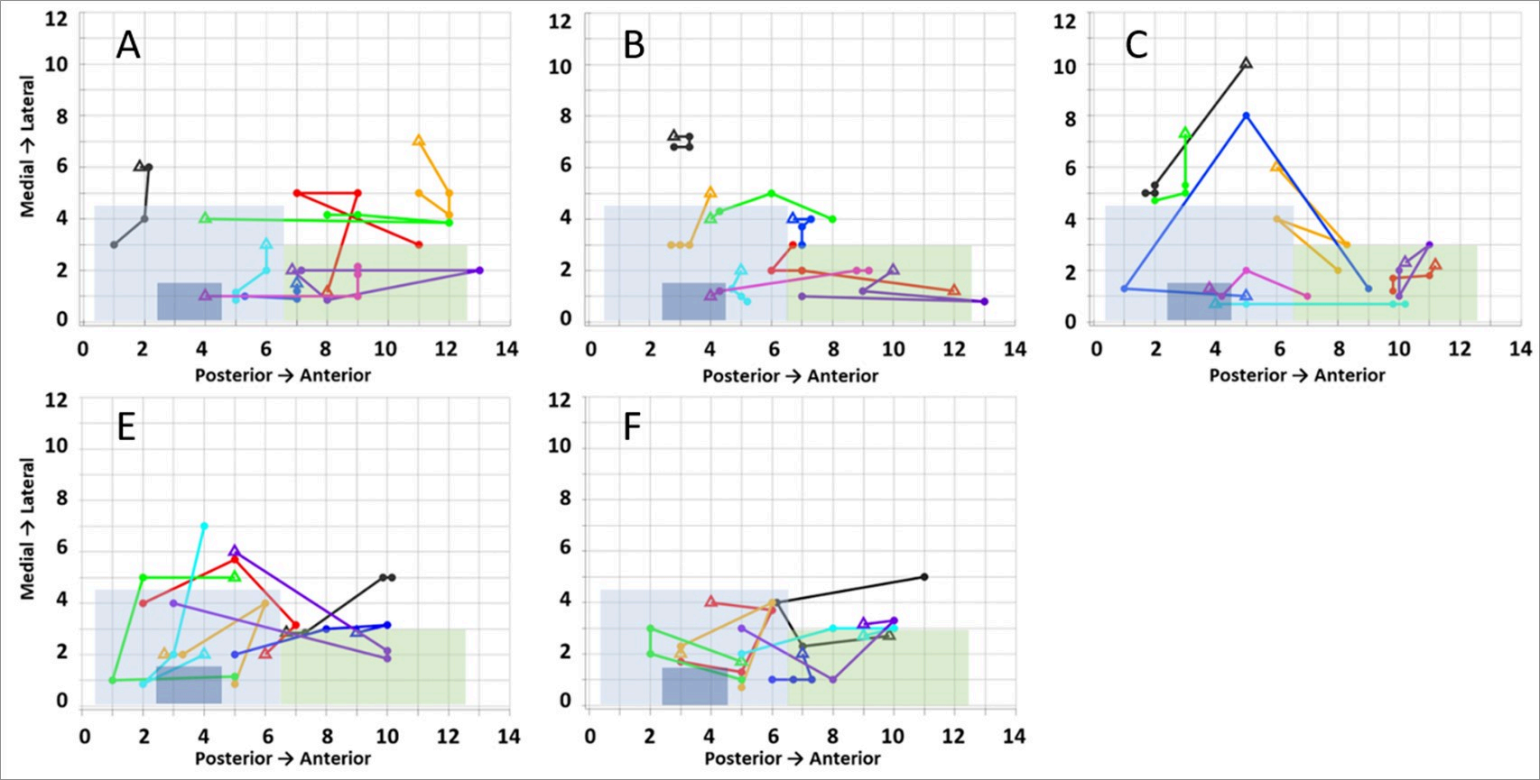
Locations of Sensation (LOS) are shown for the four therapy configurations for times of PO (Post-Op, A, E), PR (Pre-Release, B, F) and FU (Follow up in clinic, C). A-C are LOS from participants with Axonics SNM. D-E are from participants with the Medtronic Interstim Micro. The colored regions represent the anatomical locations of perianal (light blue), anal (dark blue), and genital (green) LOS. Individual patient leads are shown as different colored plots; contact configurations are sequential points along the lead. The 0−,3+ contact (triangle) and the sequential points reflect 1−,3+, 2−,0+, and 3−,0+, respectively.

LOS exhibited considerable variability within and across patients, as well as over time (Fig. 4). For PO, within-patient LOS spanned large areas (max = 12 map units, mean = 7.1+/−1.1). Similarly, this was observed at PR (max = 11 map units, mean = 7.5+/−0.7) and FU (max = 10, mean = 7.1+/−0.8). LOS across the lead also changed over time. Between PO and PR, only 12% (7 of 60) of the participants reported the same location for LOS, while participants pointed at the same coordinate or one of the eight adjacent coordinates in only 33% (20/60) of cases. The coordinate change for LOS between PR and PO ranged from zero to a maximum of 9 coordinate units. The mean absolute coordinate change was 1.3+/−1.3 units in the medial-lateral axis and 2.4+/−2.5 units in the anterior-posterior axis. Statistical tests confirmed no significant changes were present in any measures (P > 0.05 for all comparisons).

Between PR and FU time points, a similar change in LOS was observed. Participants identified the same coordinate in only 13% (4/32) of cases, with participants identifying the same coordinate or one of eight adjacent coordinates in only 22% (7/32) of cases (range:0–6). The mean absolute coordinate change from PR to FU testing for all contacts was 2.5+/−1.1 units in the medial-lateral axis and 2.5+/−1.8 units in the anterior-posterior axis. Once again, statistical tests confirmed no significant changes over time for any LOS measures (P > 0.05 for all comparisons).

## Discussion

These findings shed light on the relative utilities of using PFMT, ST, and LOS as quantitative measurements in future research. The data highlight distinctions in contacts following SNM implantation, particularly concerning PFMT, which significantly varied across electrode configurations using proximal cathode stimulation on the lead compared to more distal configurations. While a similar trend was observed in TMT, the configuration differences were absent. Notably, the discrepancy between TMT and PFMT changed along the lead’s length, with more distal contact stimulation appearing less effective and potentially limiting their clinical utility, although this was not directly explored in our study.

In contrast, ST did not exhibit significant differences across contact configurations, even when measured on the same day (PO and PR), suggesting that dressing changes and ambulation did not significantly alter ST. The decrease in overall ST from PR to follow-up (FU) was unexpected and requires further investigation. This decrease may be related to tissue changes, such as inflammation immediately following surgery or a neurostimulation-related impedance decrease that has been observed in other neuromodulation systems.^18,19^ Despite its clinical ease of measurement, ST immediately following surgery may not be as effective as PFMT in identifying quantitative differences between SNM contacts.

Surprisingly, ST was not consistently lower than PFMT, challenging the common assumption in clinical practice. In our study, the mean ST was larger than the mean PFMT, suggesting that PFMT might be a more sensitive clinical measurement than ST for many patients. Further research with larger sample sizes is needed to confirm this unanticipated outcome.

LOS measurements exhibited considerable variability, limiting their utility in neural mapping between SNM targets and sensory fields. While LOS is routinely used in programming SNM, its documentation is often insufficient post-programming.^20^ The lack of a consistent relationship between contact-specific LOS and body position suggests limited usefulness in assessing changes over time or across electrode configurations.

This study, like many small studies, has inherent limitations. Conducted as a clinical feasibility study, we intentionally avoided significantly altering established clinical practices for threshold testing. Consequently, ST and PFMT employed bipolar stimulations reflective of current medical practices. Monopolar stimulation could potentially offer better differentiation in identifying location or threshold differences across stimulation electrode configurations.^6^ Despite this, PFMT and LOS exhibited analogous differences across therapy electrode configurations, suggesting varying positions relative to the sacral nerve location. Subsequent studies should encompass a larger patient cohort and additional lead location measurements to validate these findings.

The potential for greater variability in ST or other factors may have obscured visible differences in ST measurements or LOS. While prior studies have illustrated a consistent pattern of ST along the lead length mirroring PFMT results,^19^ to our knowledge, no studies have shown statistical differences in ST along the lead’s length. One contributing factor to the absence of ST or LOS effects in our study might be the patients’ lack of familiarity with novel paresthesia shortly after implantation or the continuing effects of sedation/anesthesia following the implant procedure. Extended exposure to SNM therapy over time could offer a more stable foundation for patient perceptions and confident reporting than the initial days following therapy implantation.

Our data are further constrained by a lack of insight into which patients will exhibit favorable efficacy outcomes. The potential variability in thresholds between patients with good versus poor SNM outcomes may limit our ability to observe significant differences in ST across contacts.

The study was conducted during the COVID-19 pandemic (June to Dec 2021), introducing delays and challenges in direct clinical care. The inconsistent data collection during the follow-up visit (56% data capture vs 94% immediately post-op) likely resulted from this challenge. This also contributes to the prolonged duration between the surgical period and clinical follow-up (22+/−7d).

Finally, inconsistent implantation locations would also pose a factor impacting possible differences between therapy contacts. Overly constrained or inconsistent contact locations, comparisons across electrode configurations may not reveal the anticipated differences seen in smaller feasibility clinical testing or preclinical work. However, our team’s prior work using fluoroscopy demonstrated consistent implant locations across SNM implants.^21^ The differences in PFMT across electrode configurations we observed are similar to preclinical work,^11,12^ suggesting adequately varied contact locations in this study.

## Conclusions

These findings underscore the efficacy of PFMT as a valuable metric for distinguishing between therapy electrode configurations along recently implanted SNM leads. In contrast, ST and LOS did not exhibit the same level of utility. Notably, ST demonstrated only marginal changes over time, lacking discernible differences across therapy electrode configurations. The variability observed in LOS suggests limited utility in identifying differences across electrode configurations or tracking changes over time. While attributing changes in LOS over time to alterations in lead location is premature, this possibility remains open for further investigation. Future research should explore these physiological markers and other potential quantifications of SNM leads, ensuring continuous advancements in technology and therapeutic enhancements.

## Data Availability

Deidentified data will be made available to researchers upon reasonable request.

## Abbreviations

FU: Follow-up clinic visit period
Hz: Hertz
LOS: Location of sensation
mA: Milliamperes
PFMT: Pelvic floor motor threshold
PO: Post- Operative period
PR: Pre-release period
SNM: Sacral neuromodulation or sacral nerve stimulation
ST: Sensory threshold:smallest stimulus sensed by the individual
TMT: Toe, leg, or ankle motor threshold
V: Volts
